# Lessons learned from the coronavirus health crisis in the nordic countries

**DOI:** 10.1192/j.eurpsy.2021.117

**Published:** 2021-08-13

**Authors:** U. Valdimarsdóttir

**Affiliations:** Center Of Public Health Sciences, University of Iceland, Reykjavik, Iceland

## Abstract

**Abstract Body:**

The COVID-19 pandemic has had an unprecedented influence on the global economy and population health. Vigorous, well-designed studies with complete, long-term follow-up of high risk groups including COVID-19 patients, their families and frontline workers are imperative for a comprehensive understanding of the mental health impact of the pandemic. The Nordic-Baltic national registries and biobank resources provide a unique opportunity to gain critical insight into the interplay between mental and somatic health during the COVID-19 pandemic. The COVIDMENT consortium leverages an extensive research experience and infrastructure from ongoing collaborations between four Nordic countries and Estonia, including national registry resources (est. >24 million individuals) and new COVID-19 cohorts with questionnaire data (est. > 220.000 individuals), to significantly advance current knowledge of mental morbidity trajectories in the COVID-19 pandemic. This program will address the following specific aims: 1) The role of preexisting psychiatric disorders in subsequent risk and progression of a COVID-19 infection. 2) The impact of COVID-19 on short and long-term psychiatric sequel among COVID-19 patients, their families and frontline workers. 3) The impact of the COVID-19 pandemic on population mental health by the varying mitigating responses and corresponding COVID-19 related mortality rates across 4 Nordic countries and Estonia. These data sources and research plan, along with preliminary results will be presented.

**Disclosure:**

No significant relationships.

